# Supplementary material for: Feasibility of a pragmatic randomized adaptive clinical trial to evaluate a brief negotiational interview for harmful and hazardous alcohol use in Moshi, Tanzania
Source: PLoS One. 2023 Aug 3;18(8):e0288458. doi: 10.1371/journal.pone.0288458 (PMC10399826; doi:10.1371/journal.pone.0288458)
Supplement: S1 Table — (DOCX) [file pone.0288458.s003.docx]

**SUPPLEMENTARY INFORMATION**

**Supplementary Table S1:** Results of patient acceptability qualitative interviews.

|  | **Positive Responses** | **Negative Responses** | **Unknown** |
| --- | --- | --- | --- |
| Was this the best time of the day to receive texts, or would another be better because you were busy? | 8 | 2 | 1 |
| Do you want to continue to receive these messages? | 11 | 0 |  |
| What effect did these messages have on your behavior? | 11 | 0 |  |
| Was it a good time for the nurse-led intervention or was there a better time? | 46 | 10 |  |
| Was it the right thing to have the intervention led by the nurses? | 56 | 0 |  |
| What effect do you think this intervention had on your behavior? | 56 | 0 |  |
