# Supplementary material for: Feasibility of a pragmatic randomized adaptive clinical trial to evaluate a brief negotiational interview for harmful and hazardous alcohol use in Moshi, Tanzania
Source: PLoS One. 2023 Aug 3;18(8):e0288458. doi: 10.1371/journal.pone.0288458 (PMC10399826; doi:10.1371/journal.pone.0288458)
Supplement: Attachment — Submitted filename: rebuttal letter second revision.docx [file pone.0288458.s004.docx]

To the in-house editors of PloS One:

Thank you for these comments. Here are our detailed responses:
1. Please note that you must upload a completed CONSORT flowchart as figure 1 of your manuscript. Blank copies of this document and information regarding CONSORT can be found via the following link: [https://urldefense.com/v3/__http://www.consort-statement.org/__;!!OToaGQ!4JeqKMT5-Y8GSAeQujzFw1hOm6wIirpQzkkjf6ARulUz0H-dr2KMqisgmegA_WYLwKH2Tg$](https://urldefense.com/v3/__http:/www.consort-statement.org/__;!!OToaGQ!4JeqKMT5-Y8GSAeQujzFw1hOm6wIirpQzkkjf6ARulUz0H-dr2KMqisgmegA_WYLwKH2Tg$) .

*Author Response: We added the workflow figure representing the study design. We would like to add that while our study shares several characteristics of a trial, but it is still a feasibility study. As a pilot evaluation of the intervention proposed to be part of a larger trial, the feasibility study has special features as a reduced sample, and the lack of statistical power and sample size calculations, and limited preliminary outcome analysis. Thus, we kindly like to ask to keep these differences in mind when assessing the manuscript.*

2. Please note that you must upload a completed CONSORT checklist as a supporting information file. Blank copies of this document and information regarding CONSORT can be found via the following link: [https://urldefense.com/v3/__http://www.consort-statement.org/__;!!OToaGQ!4JeqKMT5-Y8GSAeQujzFw1hOm6wIirpQzkkjf6ARulUz0H-dr2KMqisgmegA_WYLwKH2Tg$](https://urldefense.com/v3/__http:/www.consort-statement.org/__;!!OToaGQ!4JeqKMT5-Y8GSAeQujzFw1hOm6wIirpQzkkjf6ARulUz0H-dr2KMqisgmegA_WYLwKH2Tg$) . If your clinical trial uses a non-randomized design, you may wish to submit a TREND checklist ([https://urldefense.com/v3/__http://www.cdc.gov/trendstatement__;!!OToaGQ!4JeqKMT5-Y8GSAeQujzFw1hOm6wIirpQzkkjf6ARulUz0H-dr2KMqisgmegA_Wai_m4Qrg$](https://urldefense.com/v3/__http:/www.cdc.gov/trendstatement__;!!OToaGQ!4JeqKMT5-Y8GSAeQujzFw1hOm6wIirpQzkkjf6ARulUz0H-dr2KMqisgmegA_Wai_m4Qrg$) ), in place of the CONSORT checklist.

*Author Response: We filled the CONSORT protocol checklist as requested, and we are submitting this document as part of the revision process.*

3. Please upload a copy of your trial study protocol as a supporting information file. By the study protocol, we mean the complete and detailed plan for the conduct and analysis of the trial that the ethics committee approved before the trial began. Please send this in the original language. If this is in a language other than English, please also provide a translation. Please detail any deviations from this study protocol in the Methods section of your manuscript. Your study protocol will be made available to the editors and reviewers, and will be published as supporting information with your manuscript if accepted for publication. (If you do not agree to this, we will not be able to publish your manuscript). If you have formally published a study protocol for your trial in a journal then you should cite this in your manuscript, but you still need to send us the original document.

*Author Response: Thanks for your comment. We upload the study protocol registered at https://clinicaltrials.gov/ct2/show/NCT02828267?term=NCT02828267&draw=2&rank=1. The study protocol was approved before the study began.*

Catherine A. Staton, MD MScGH

Associate Professor of Emergency Medicine and Global Health

EM Director, Hubert Yeargan Global Health Residency and Fellowship Pathway

Director, Global Emergency Medicine Innovation and Implementation (GEMINI) Research Lab

Vice Chief of Faculty Development, Division of Emergency Medicine,

Department of Surgery, Duke University School of Medicine, Duke Global Health Institute
