# Supplementary material for: Feasibility of a pragmatic randomized adaptive clinical trial to evaluate a brief negotiational interview for harmful and hazardous alcohol use in Moshi, Tanzania
Source: PLoS One. 2023 Aug 3;18(8):e0288458. doi: 10.1371/journal.pone.0288458 (PMC10399826; doi:10.1371/journal.pone.0288458)
Supplement: Attachment — Submitted filename: BNI_Feasibility_PLOS_One_rebuttal letter.docx [file pone.0288458.s005.docx]

To PLoS One Editors,

Thank you so much for the opportunity to revise this manuscript. As instructed, we have created a point-by-point rebuttal to each editor and reviewer point, a marked-up copy, and an unmarked-up version.

Journal Requirements:

1. Please ensure that your manuscript meets PLOS ONE's style requirements, including those for file naming. The PLOS ONE style templates can be found at

[https://journals.plos.org/plosone/s/file?id=wjVg/PLOSOne_formatting_sample_main_body.pdf](https://urldefense.com/v3/__https:/journals.plos.org/plosone/s/file?id=wjVg*PLOSOne_formatting_sample_main_body.pdf__;Lw!!OToaGQ!vzIx-aAxP6WNvvuFrgnJNJ5h-RpvK09WygZVJ63Er2-Ks3knK5F9x3XJiRY39kPs7oI95C9tjaPpjMogNDrD0A$) and

[https://journals.plos.org/plosone/s/file?id=ba62/PLOSOne_formatting_sample_title_authors_affiliations.pdf](https://urldefense.com/v3/__https:/journals.plos.org/plosone/s/file?id=ba62*PLOSOne_formatting_sample_title_authors_affiliations.pdf__;Lw!!OToaGQ!vzIx-aAxP6WNvvuFrgnJNJ5h-RpvK09WygZVJ63Er2-Ks3knK5F9x3XJiRY39kPs7oI95C9tjaPpjMrotAW2tA$)

**We will do this, thank you.**

2. We note that the grant information you provided in the ‘Funding Information’ and ‘Financial Disclosure’ sections do not match.

When you resubmit, please ensure that you provide the correct grant numbers for the awards you received for your study in the ‘Funding Information’ section.

**We will do this, thank you.**

3. Your ethics statement should only appear in the Methods section of your manuscript. If your ethics statement is written in any section besides the Methods, please delete it from any other section.

**We will do this, thank you.**

4. Please include captions for your Supporting Information files at the end of your manuscript, and update any in-text citations to match accordingly. Please see our Supporting Information guidelines for more information: [http://journals.plos.org/plosone/s/supporting-information.](https://urldefense.com/v3/__http:/journals.plos.org/plosone/s/supporting-information.__;!!OToaGQ!vzIx-aAxP6WNvvuFrgnJNJ5h-RpvK09WygZVJ63Er2-Ks3knK5F9x3XJiRY39kPs7oI95C9tjaPpjMp67vRe6A$)

**We will do this, thank you.**

Points raised during the review process:

This submission has been evaluated by three reviewers whose comments are included below. Please address the reviewers' comments (except Reviewer 1, point 1) through revisions to your manuscript and in response to the Reviewer's document.

Reviewer 1's first comment raised, "Authors have mentioned that the aim of the study is to assess the feasibility however, the title of the study mentions it as a randomized controlled trial." As your title identifies the study as a feasibility study, you can disregard this comment.

**Thank you.**

You addressed a sample size calculation query in your Response to Reviewers section, and this issue was again raised for the current submission by Reviewer 2. From your response to Reviewers' comments it is my understanding that you did not do a formal sample size calculation for this study. In the Sample Size section of the Methods, please clarify the approach taken in deciding upon the sample size (i.e. the rationale for the group sizes used), and transparently state whether a formal sample size calculation was not performed. Also, please discuss this issue in the Limitations section in the context of the references cited by Reviewer 2 for the prior submission and Reviewer 2 for the current submission.

**Yes, as a feasibility study we should not and are not powered to perform an evaluation of a hypothesis/ or effectiveness testing. A sentence on this was added in the sample size section of the methods and addressed in the limitations.**

In the Response to Reviewers, you explained that there were differences between the original approved study protocol, manuscript, and Clinical Trials registry for this study. Please add a section to the Methods section that discusses any changes from the original approved protocol; explains any discrepancies between the manuscript, original protocol, and registry; and notes if/when any changes were approved by the IRB.

**Thank you so much for this suggestion. A paragraph was added describing this.**

Reviewer Comments:

Reviewer #1: Dear Author,

The manuscript has covered feasibility of important intervention in low resource setting. There are some major and minor comments which need to be addressed before it can be considered for the publication.

1. Major comments: Authors have mentioned that the aim of the study is to assess the feasibility however the title of study mentions it as randomized controlled trial which is inherently supposed to measure effectiveness of the intervention vis a vis control group. So authors must do statistical analysis to see if there is any significant or non significant differences in outcome measures using non-parametric tests since the sample size may not be enough for parametric tests. Otherwise it would be wrong to refer the present manuscript as RCT. The paper can be best described as feasibility study and not RCT. So please update results sections to compare outcome measures accordingly. The findings should also be discussed later.

**Thank you for this feedback. This is a feasibility study and therefore are reporting the feasibility on how to conduct this trial. Our future trial will report sample size and statistical analysis for our efficacy testing analyses.**

2. Results section does not mention about findings of 15 questions in a post-

treatment survey/interview and 5 questions about satisfaction

**Thank you for this feedback. Responses to these questions have been included in the results according to the topic of feasibility.**

3. The trial has higher retention rate, the reason for higher retention rate should be highlighted (e.g. home visits by investigators, financial incentives given to the project staff and number of times follow up calls were attempted). All these issues are important when intervention is used in the clinical setting.

**Thank you for this feedback. Yes, we have a high retention rate in our studies due to our very proactive process in following our patients. This is a locally relevant process which we have not specifically identified because it is very site dependent. We have addressed the activities supporting our high retention rate in the discussion section.**

4. The authors have not mentioned what treatment was offered for patients with alcohol use disorders, any psychiatrist consultation was sent ? referred to outpatient department for management of the problem?

**Thank you for this feedback. Our treatment was a standard SBIRT with screening, brief intervention with our adapted version of the BNI called the PPKAY: Punguza Pombe Kwa Afya Yako. Only method of treatment at the time was the screening, brief intervention.**

5. The timing of intervention is ranged between 5 minutes to 30 minutes. It is important to mention average time of intervention. As this raises question about fidelity of intervention. It will be good to mention number of nursing professionals who were involved in delivery of intervention, any family members involved as some patient may be brought by law enforcement agencies while some may brought by family members. Involvement of family members are likely to impact outcome measures. Reasons--

**Thank you for this feedback. Fidelity of this intervention was measured by reviewing audio-recorded versions of the intervention and rating it on the BI assessment checklist to ensure nurses were completing desired components of the intervention. Feedback was then provided to the nurses for future interventions. Fidelity was not measured on time given 5-30 minute intervention was dependent on patient responses, not questions asked.**

**An update was made in the paper to note three research nurses performed the intervention to ensure consistency. No family members were involved at this point of the feasibility trial due to stigma. Prisoners were unable to participate.**

6. Discussion section need to be expanded to discuss the findings in reference to available literature from the region. Also clinical implications of the study if any.

**Thank you for this feedback. A sentence was added to include a systematic review of brief interventions in LMIC to connect to literature from the region. No clinical implications since the study is is a feasibility pilot. The implication of this study is that the trial seems feasible and should be performed.**

Reviewer #2:

The manuscript explores the feasibility of a pragmatic randomized adaptive controlled trial of the PPKAY intervention among subjects recruited in Tanzania. The study objectives are clearly stated; the authors proposed a block-randomized controlled trial. In the future, the effectiveness of the intervention will be assessed via a pragmatic randomized adaptive trial. My comments are as follows:

1. I still think a sample size/power statement would make the approach more rigorous. Just stating that someone included 10 participants in each arm do not make a strong case. Samples sizes for pilot/feasibility studies are requested; see below:

[https://www.rds-london.nihr.ac.uk/resources/justify-sample-size-for-a-feasibility-study/](https://urldefense.com/v3/__https:/www.rds-london.nihr.ac.uk/resources/justify-sample-size-for-a-feasibility-study/__;!!OToaGQ!vzIx-aAxP6WNvvuFrgnJNJ5h-RpvK09WygZVJ63Er2-Ks3knK5F9x3XJiRY39kPs7oI95C9tjaPpjMpLCB0bhg$)

Also, the 3-stage design as proposed is not so trivial.

**Thank you for this feedback. We included 10 participants in each allocated arm to ensure at least 20 participants at each stage. The study included a total of 70 participants (table 2). We used this method to replicate the average potential enrollment per week. Ensured this was addressed in sample size section.**

Reviewer #3:

1. The data appear to support the conclusions regarding the feasibility to conduct a large scale trial. What stood out for me was just how well received this feasibility study was to both patients and providers. When I saw in the limitations that the study PI was the providers boss then I think that may explain the positive views from the staff and is therefore quite a large limitation. I think this would seriously need consideration in the planning of a future trial as staff may be less receptive in other settings. Especially if it can take 30 minutes to deliver the intervention in some cases. I think the paper should include some discussion of challenges that may occur in a large scale trial that would take place across multiple settings as it does come across as overly positive.

**Thank you for this feedback. A sentence was added to the paper to discuss the potential impact of familiarity on positivity and consider including champions to address widespread dissemination challenges and maintain positivity.**

2. The statistics appear sound, but as I am not a trial statistician I think another reviewer may be better placed to comment here.

**Thank you.**

3. The author stated that the data can not be made fully available due to privacy concerns. Perhaps some further information could be provided here, is it not possible to anonymize the datakjh

**Thank you for this comment. The data is available with appropriate ethical consideration. Data can be obtained through regulatory Tanzanian data transfer .**

4. The article is well written and I like how it is organized around the RE_AIM framework. A couple of points: Page 38/68 (page 8 of manuscript) - Is there a reference for where you adapted the tools culturally using data? It would be good to include the reference here again if so

**Thank you for this comment. The paper is currently under review in another journal, but a reference was added.**

Page39/68 (page 9) - How did they train nurses in the intervention delivery? I noticed that as part of fidelity checks, nurses were evaluated on how they delivered the intervention according to protocol and that the discussion reports that they were satisfied with the training - but it would be good to see in the methods how you trained them. Although brief, this is a behavioral change intervention that would require a certain standard of training that ideally should be described in the paper for replication purposes and also evaluated in order to see if the nurses are capable of delivering the intervention to protocol.

**Thank you for this feedback. Information on how nurses were trained was added.**

P9 - "Unusual care" heading - did you mean Usual care?!

**Thank you for this feedback. The heading was changed to usual care.**

p10 - Is home brew common in this region? If it is then this may make it difficult to estimate consumption in a larger study.

**Thank you for this comment. Homebrew is common in this region, and we have researched to determine standard drink size in the region. We can estimate a culturally relevant amount at the same and different time frames. Ensured this was addressed in the paper.**

p12 - Table 3 - I think there is a typo here and it should say Farmer instead of Famer

**Thank you for this feedback. Famer was changed to Farmer.**
